# Tree Species Composition and Forest Community Types along Environmental Gradients in Htamanthi Wildlife Sanctuary, Myanmar: Implications for Action Prioritization in Conservation

**DOI:** 10.3390/plants11162180

**Published:** 2022-08-22

**Authors:** Myo Min Latt, Byung Bae Park

**Affiliations:** 1 Department of Environment and Forest Resources, Chungnam National University, Daejeon 34134, Korea; 2 Department of Natural Resources Management, University of Forestry and Environmental Science, Yezin 15013, Myanmar

**Keywords:** edaphic factors, forest communities, Htamanthi, Myanmar, soil hardness, species diversity, topographic factors

## Abstract

The identification of forest community types is essential for prioritizing choices and targets in species and community conservation purposes amid climate change impacts on forest community dynamics. Here, we determined the tree species composition, species diversity, and the forest community types across contrasting topographic and edaphic conditions in Htamanthi Wildlife Sanctuary (HWS), Myanmar. All tree species with diameter at breast height (DBH) ≥10 cm were recorded in 66 plots (625 m^2^), from which the species diversity, density, frequency, dominance, and importance value (IV) of each tree species were measured. The soil hardness (Hd), bulk density (BD), moisture content (MC), organic matter content (OM), texture, pH, total N, and available P, K, Ca, Na, and Mg concentrations were also analyzed. The elevation (ELV) and slope (SLP) were also measured as the topographic factors. Cluster analysis resulted in five distinct forest communities and the soil Ca, Mg, clay proportion, soil hardness, and elevation were the major influencing factors. The species diversity in HWS ranged from low to very high relative values, with 209 tree species belonging to 119 genera and 55 families. Identification of these community types and understanding the diversity levels and major factors influencing the community structure may play a key role in the planning, prioritization, and implementation of species and community conservation strategies amid the unpredictable impacts of climate change on forest community dynamics.

## 1. Introduction

Climate change has been altering the structure and functions of forest ecosystems worldwide. The unprecedented rise in global temperature may lead to the modification of various environmental variables and, thus, may shift in species composition and alterations in forest community structure at the local scale. Even minor changes in microclimatic conditions with elevation may also result in unusual changes in local diversity [1,2]. Environmental gradients, which refer to variations in site characteristics (i.e., edaphic, climatic, and physiographic variables), influence the patterns of tree species composition and distribution of forest communities [3]. Many interacting biotic and abiotic factors, including changes in elevation, slope, soil properties, and site index, affect species richness [4]. Climatic factors vary with elevation and exert a strong influence on plant distribution across ecosystems [5]. Thus, understanding the pattern of tree species composition and the types of forest communities across contrasting environmental conditions may play a key role in the planning, prioritization, and implementation of species and community conservation strategies amid the unpredictable impacts of climate change on forest community dynamics.

The pattern in tree species composition and forest community may vary among groups of plants and from one area to another due to differences in resource availability, plant life-history traits, and adaptive strategies of plants, as influenced by the prevailing environmental conditions [6,7]. Soil nutrient and moisture availability, for instance, can influence natural regeneration, seedling establishment, and species dominance depending on elevation [8,9,10]. Climatic factors, such as air temperature and precipitation, have an interactive role in the effects of soil nutrients on species dominance [6]. A study reported that a high soil nutrient supply, particularly nitrogen and phosphorus, can lead to a lower species richness and high soil moisture can result in a higher species richness [11]. It has also been suggested that soil pH exerts a strong influence on species composition at the local scale [12] by limiting the number of species that can adapt to the extreme ends of the pH gradient [13]. While numerous studies have focused on large geographical areas, there are fewer studies about tree species composition and distribution of forest communities along environmental gradients conducted in smaller landscapes or mountains. Because global changes in environmental conditions have a direct impact on local ecological systems, localized studies may contribute to global biodiversity conservation programs through prioritization of habitats and conservation strategies.

Htamanthi Wildlife Sanctuary (HWS), an ASEAN heritage park in Myanmar and probably the largest contiguous forest landscape in Asia, lies in a transition zone of three biodiversity hotspots [14]. The sanctuary is globally important because of its proximity to the Northern Forest Complex (NFC), one of the largest remaining contiguous forests in Southeast Asia. Despite the ecological importance of HWS, most earlier studies dealt with faunal resources and less attention was given to the floral resources, particularly trees. By knowing the patterns of tree species composition and types of forest communities along environmental gradients in this biodiversity hotspot, we could advance our understanding of the local forest communities and how the environmental variables affect these communities.

The identification of forest community types can play a key role in prioritizing conservation choices in biodiversity hotspots since nature-based solutions are emerging as an integrated approach to conserving biodiversity and ecosystem services [15]. One of the practical strategies in biodiversity conservation is the identification of priority areas featuring exceptional need for immediate conservation, particularly biodiverse areas experiencing high degree of habitat loss and risks [16]. This is because one of the major challenges commonly encountered by conservation practitioners is how to efficiently allocate limited resources to many focal ecosystems or forest plant communities needing conservation [17]. Thus, information on forest community types in Htamanthi Wildlife Sanctuary can subsequently be useful for the development of more efficient ecosystem-based conservation approaches amid limited resources.

Consequently, we determined the tree species composition, species diversity, and the forest community types along environmental gradients in HWS. The role of the environmental factors in shaping forest communities in HWS remains poorly studied. Identification of species and community structure is very relevant to understanding the status of tree populations, natural regeneration for species, and community conservation purposes amid climate change impacts on forest community dynamics [18].

## 2. Results

### 2.1. Forest Community Assemblages

**Figure 1:**
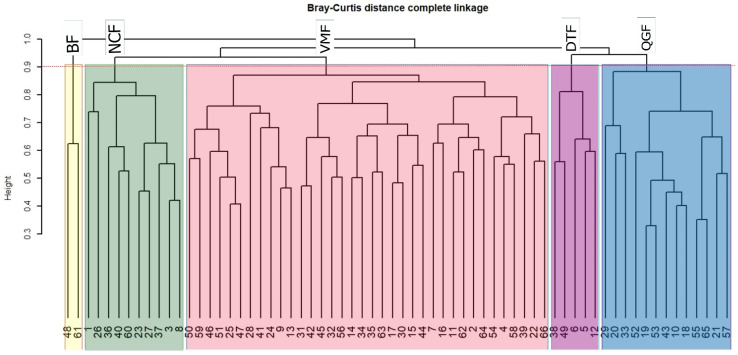
Dendrogram of all plots based on Bray–Curtis Distance complete linkage showing the five distinct forest communities of evergreen forest ecosystem in Htamanthi Wildlife Sanctuary in Myanmar. Forest communities: Bamboo forest (BF), *Vatica maingayi* forest (VMF), *Quercus glauca* forest (QGF), *Nothaphoebe condensa* forest (NCF), and *Diospyros toposia* forest (DTF).

In this study, the Bray–Curtis dissimilarity cluster analysis grouped the evergreen forest in Htamanthi Wildlife Sanctuary into five distinct forest communities (Figure 1). The biggest group is *Vatica maingayi* forest (VMF) with 36 plots, *Quercus glauca* forest (QGF), *Nothaphoebe condensa* forest (NCF), and *Diospyros toposia* forest (DTF) are intermediate, with 5–13 plots, and the smallest group is the bamboo forest (BF), with only two plots.

### 2.2. Species Diversity and Importance Values among Forest Communities

In this study, we identified 209 tree species belonging to 119 genera and 55 families across the five forest communities (Table S1). The VMF and BF forest communities have very high (3.965) and very low (0.086) Shannon (H) diversity values, respectively (Table 1). The same pattern was observed in Evenness (J’) values for these two forest communities. The QGF has a high diversity value of H = 3.25. The other forest communities (NCF and DTF) have moderate H values (2.659–2.807). The VMF is a major forest community composed of a higher number of species, genera and family, followed by NCF, QGF, DTF, and BF (Table S1). In terms of IV, the most dominant tree species is *Vatica maingayi* in VMF, *Nothaphoebe condensa* in NCF, *Quercus glauca* in QGF, and *Diospyros toposia* in DTF (Table 1 and Table S1).

### 2.3. Variations in Topography and Soil Characteristics among Forest Communities

There was a significant difference in the elevation and slope across the four forest communities (Table 2). The highest elevation was in DFT, intermediate in NCF, and the lowest value was found in both QGF and VMF. In terms of slope, DFT is the steepest among the forest communities.

At 0–15 cm soil depth, the MC, sand, and clay proportions varied significantly across forest communities (Table 3 and Table S2). The soil moisture (MC) in NCF and VMF were higher than DTF and QGF. Further, DTF and QGF have similarly high sand and clay proportions compared the other forest communities. At 15–30 cm soil depth, the Hd, MC, and clay proportion were significantly different across sites. Both DTF and NCF have a higher Hd than QGF or VMF. The DTF and QG have similarly higher MC than the other forest community types and a similar pattern was observed in clay proportions.

In terms of soil chemical characteristics, the available phosphorus (AP) was higher in DDT than those in the other forest communities at 0–15 cm soil depth (Table 4 and Table S3). An almost similar pattern was observed in Ca and Mg concentrations for both DDT and NCF. At 0–15 cm soil depth, the AP, Ca, and Mg were also significantly higher in DDT and/or NCF than the other forest community types (Table 4).

### 2.4. Redundancy Analysis (RDA) Biplot and Important Topographic and Edaphic Variables

**Figure 2:**
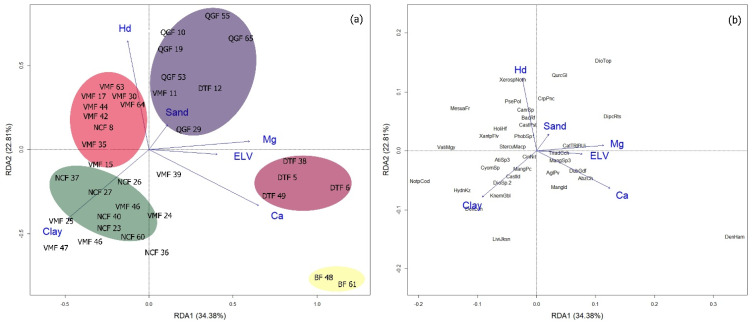
Redundancy analysis (RDA) biplot showing the (**a**) relationship among environmental variables and forest communities and (**b**) relationship among environmental variables and dominant tree species at 0–15 cm soil depth in Htamanthi Wildlife Sanctuary. Abbreviations: ELV—elevation, Hd—soil hardness, Mg—extractable magnesium, Ca—extractable calcium; forest communities: *Vatica maingayi* forest (VMF), *Quercus glauca* forest (QGF), *Nothaphoebe condensa* forest (NCF), and *Diospyros toposia* forest (DTF). The number after the abbreviated name of the forest community in panel (**a**) indicates the plot number. In panel (**b**), the black lowercase letters indicate the abbreviated scientific names of dominant tree species (Table S1).

To provide an additional quantification of the proportion of the variance in the data set and confirm that environmental variables influence the structure and classification of forest communities, we performed an RDA. At 0–15 cm soil depths, the relationship among the variables is significant (*p* = 0.001, Table S4). The first RDA axis and second axis explain 34.38% and 22.81% of the total variance, respectively (Figure 2). RDA1 is strongly and positively associated with ELV, Ca, and Mg, which are well represented in DTF community or *D. toposia* (DioTop)-dominated forests (Figure 2b). RDA1 is also strongly and negatively associated with clay proportion in VMF. RDA2 was highly associated with Hd in QGF community or *Q. glauca* (QurcGl)-dominated forests (Figure 2b).

**Figure 3:**
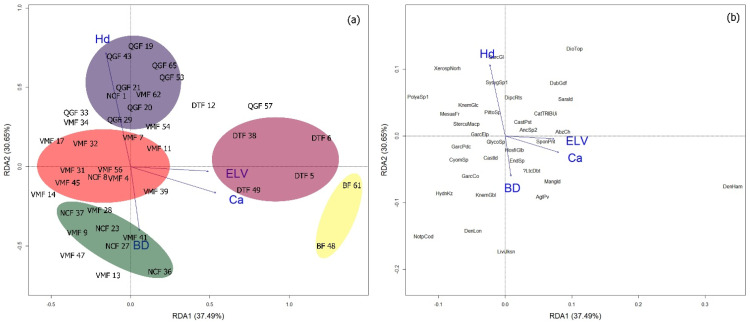
Redundancy analysis (RDA) biplot showing the (**a**) relationship among environmental variables and forest communities and (**b**) relationship among environmental variables and dominant tree species at 15–30 cm soil depth in Htamanthi Wildlife Sanctuary in Myanmar. Abbreviations: ELV—elevation, Hd—soil hardness, Mg—extractable magnesium, Ca—extractable calcium; forest communities: *Vatica maingayi* forest (VMF), *Quercus glauca* forest (QGF), *Nothaphoebe condensa* forest (NCF), and *Diospyros toposia* forest (DTF). The number after the abbreviated name of the forest community in panel (**a**) indicates the plot number. In panel (**b**), the black lowercase letters indicate the abbreviated scientific names of dominant tree species (Table S1).

At 15–30 cm soil depth, the relationship among topographic factors, the properties of soil at 15–30 cm soil depths, and ecologically important tree species and five forest communities is significant (*p* = 0.001, Table S4). The first two RDA axes accounted for 68.14% of the variations in the data set (Figure 3). Specifically, RDA1 accounted for 37.49% of the variation and was highly and positively related to Ca and ELV in DTF community. RDA2 accounted for 30.65% of the variation and was positively related to Hd in QGF and negatively to BD in VMF community.

Further, the forward selection approach revealed Ca (*p* = 0.001), clay proportion (*p* = 0.01), and Hd (*p* = 0.05) as the most contributive factors affecting the composition of tree species and classification of forest communities in the study area at 0–15 cm soil depth (Table S5). At 15–30 cm soil depth, the ELV, Hd, and Ca were the major factors affecting the classification of forest communities in Htamanthi Wildlife Sanctuary (Table S6).

## 3. Discussion

In this study, the identified forest community types showed a significant variation in their tree species richness, diversity, and evenness. Results revealed that the VMF and QGF have high to very high Shannon (H) diversity and Evenness (J’) values compared with the other community types. This variation could be ascribed to the differences in the communities in terms of topographic and edaphic factors through their influence on the dispersion behavior of tree species [19]. From a community ecology perspective, results suggest that VMF and QGF community types are more stable and resilient to natural disturbances and accelerating rates of environmental change than the other types of forest communities. Under a range of environmental disruptions that could occur in the future amid climate change, VMF and QGF may be able to provide essential forest ecosystem functions for the region. However, the impacts of environmental disruptions on ecosystem functions depend on the characteristics of forest communities that are related to resilience, including species diversity, relative abundance, and ability of the dominant species to resist regime shifts and recover functions following disturbance [20]. Here, the RDA biplot shows that the clay proportion and soil bulk density (BD) are highly associated with VMF, suggesting that any significant disturbance in these factors could potentially influence the community structure and functions. This is supported by the presence of the dominant species, *V. maingayi*, which is a dipterocarp tree species, typical of clay soil in lowland dipterocarp forests [21]. Two of the important factors influencing clay formation are the effects of soil moisture and temperature on weathering processes, which are the important aspects of regional climate change impacts on soil [22,23]. Similarly, the elevated global temperature may increase soil BD via climate change stresses (e.g., drought) and forest management activities [24]. Thus, our results suggests that, as climate change worsens, monitoring the changes in clay formation or weathering processes and soil bulk density is essential for the maintenance of VMF community structure and functions. Dealing with these two major factors influencing VMF community may represent a crucial step for determining priorities in plant community conservation.

The RDA biplot shows that the QGF community is highly associated with soil hardness (Hd), which is a good indicator of soil compaction and strength. This result suggests that HD may be an important factor controlling the plant community in QGF through either detrimental effects on subsequent natural regeneration in the area or enhancement of plant growth and accumulation of soil nutrients. The growth and survival of tree saplings can significantly decrease in compacted soil [25] through a reduction in root cell size, root penetration and, thus, acquisition of essential elements [26]. This may explain the observed negative correlation between Hd and Ca availability in soil. Contrarily, a study found that moderate-level soil compaction improved the plant uptake of P, K, Mg, Ca, and other elements, suggesting that soil compaction effects may vary depending on the severity [26]. Moreover, because soil hardness or soil compaction increases with decreasing soil moisture, the QGF may be more vulnerable to drought than the other forest communities, especially in years with little rainfall. The abundance of the slow-growing and drought-tolerant species, *Q. glauca,* can further explain the high association between Hd and the QGF community. As a drought-tolerant species, *Q. glauca* can thrive and flourish in low-resource and harsh environments, including those which are poorly drained and have tight soil spaces [27]. It has been projected that climate change will increase the frequency of drought and, thus, the effects on the plant community structure [28]. Results suggest that it is necessary to manage and prevent the causes of either surface or subsurface hardness constraints in the QGF community.

The DTF and NCF have moderate diversity values. This can be attributed to the possible effects of elevation (ELV) and soil nutrient availability, particularly Ca and Mg. The RDA biplot shows that the DTF community is highly associated with ELV, Ca, and Mg. The DTF has the highest ELV among the identified forest communities and the value is nearly similar to that of NCF. Results support the findings of Ohdo and Takahashi [29], who reported that the number of tree species decreased at high elevations and the pattern was attributed to soil nutrient availability. In the Himalaya Mountains, it was observed that soil nutrients (N, P, K, and Mg) decreased significantly with elevation [30]. In this study, the concentrations of Ca and Mg were higher in both DTF and NCF communities compared with the other community types. Higher elevation in the two communities may have influenced the concentration of exchangeable cations present in O and/or A horizons, i.e., Ca and Mg may have increased as elevation increased. Such an increase may be due to the decline in tree species composition as elevation increases. Fewer plants may decrease the demand for Ca and Mg ions, resulting in a greater number of ions left in the soil. This can be supported by the presence of the dominant species, *D. toposia,* which is typical of undisturbed forests with high amounts of nutrients [31]. *D. toposia* is also an evergreen tree species and evergreen plants usually exhibit a more conservative strategy, resulting in greater resource conservation.

## 4. Materials and Methods

### 4.1. Study Site and Sampling Method

**Figure 4:**
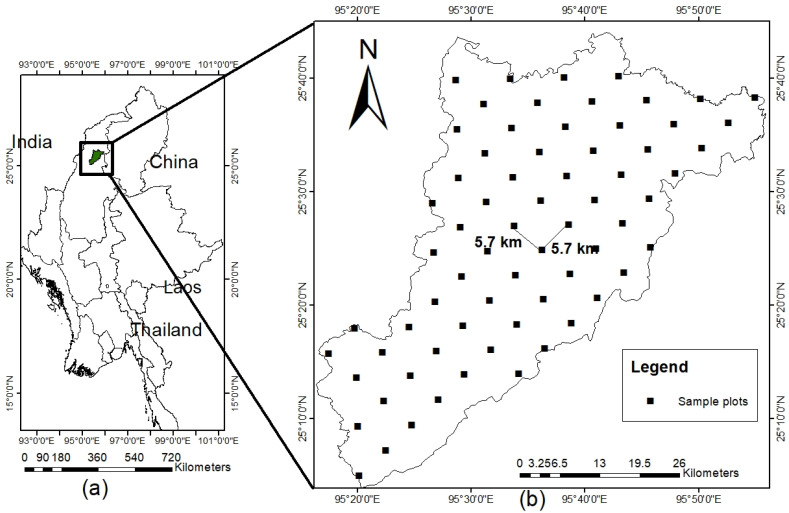
(**a**) Location of study site (Htamanthi Wildlife Sanctuary) and (**b**) distribution of sample plots.

The study was conducted in Htamanthi Wildlife Sanctuary (HWS, 25°45′52″ N to 25°45′15″ N, 95°16′55″ E to 95° 56′ 55″ E), which is a part of Himalaya biodiversity hotspot area in the northern part of Myanmar (Figure 4a). It was declared a protected area in 1974 with the extent of 215,073 hectares and, recently, it was declared the newest ASEAN heritage park. It is a dense forest comprising different habitats, including evergreen and semi-evergreen forests, swamp forests, and upper mixed dry deciduous forests. Some unique and ecologically important flora can also be found in the area, including *Tectona grandis, Xylia xylocarpa,* and *Shorea robusta.* The heritage park is generally pristine because of the minimal disturbance and absence of tourism-related activities. The annual air temperature and annual rainfall in the study site ranged from 17.7 to 34.3 °C and 343 mm, respectively, based on the 10-year climatic data from the local meteorological station near the HWS. The soil types in the study area are generally yellowish red, derived from acrisol parent material.

Sixty-six plots having a size of 25 m × 25 m were established in the study site by employing a systematic sampling (Figure 4b). The distance between plots was 5.7 km × 5.7 km. All adult trees with diameter at breast height (DBH) ≥ 10 cm were recorded in 25 m × 25 plots. The vegetative inventory was conducted from November to February in 2019 and 2021.

### 4.2. Determination of Topographic and Edaphic Variables

The physiographical variables, namely, geographic coordinates, elevation (ELV), slope (SLP), and aspect (ASP) were recorded for each plot using a GPS (Garmin GPSMAP 62s) device. The ALOS PALSAR RTC DEM (12.5 m) images were obtained from the Alaska Satellite Facility (UAF). Thereafter, ELV, SLP, and ASP of the sample plots were extracted from images and processed using ArcMap software (version 10.8, Esri, CA, USA).

Soil samples were collected from the three pre-determined points (10 m apart) in the 25 × 25 m plot using soil core sampler (5.0 cm in diameter). Three samples (c.a. 100 g) were collected from each soil depth (0–15 m and 15–30 cm) in each point and then composited into one bag. There was a total of 132 bags of soil (i.e., 1 bag × 2 soil depths × 66 plots = 132). The soil hardness (Hd) was measured at each soil depth and plot using a penetrometer. The soil bulk density (BD), the content of moisture content (MC), organic matter composition (OM), texture (sand, silt, clay), and seven chemical properties of soil; pH, total N, Available P, exchangeable K, Ca, Na, and Mg, were also analyzed using c.a. 100 g of composited soil sample at the Forest Soil Laboratory, Forest Research Institute (FRI), Yezin, Myanmar.

### 4.3. Data and Statistical Analyses

In this study, the species density, dominance, and frequency were calculated for all trees identified in each plot using the formulae in Table 5. The importance value (IV in %) of tree species encountered in the sampled plots were computed by obtaining the summation of the relative values of stem density, dominance, and frequency. The IV measures a given species’ dominance in a forest area based on species and stand structure. In this study, the IV was determined to describe the ecological significance of the species in HWS.

The Bray–Curtis cluster analysis was performed in RStudio software (version 4.2.0) using the vegan package to classify the vegetation into distinct forest community types. The similarity and dissimilarity among forest community types were determined using the Jaccard similarity index based on the species abundance data. The Jaccard’s similarity index formula is: J (i, j) = a/(a + b + c), where a = number of species in common between the communities; b = number of species unique to the first community; c = number of species unique to the second community.

The species richness (S), evenness (J), and Shannon–Wiener diversity index (H’) were computed using the R software. The Shannon diversity index (H’) was computed from the equation: H = −Σpi × ln(pi), where H is Shannon–Wiener diversity index, pi is the proportion of individual tree species.

Kruskal–Wallis test was applied to assess the significant differences in topographic and physico-chemical characteristics across the different forest communities. Pairwise comparisons among the different forest communities were conducted by Dunn–Bonferroni post hoc test method.

Here, redundancy analysis (RDA) ordination was used in describing the relationship between forest communities, topographic factors, and physico-chemical properties of soil. The global model test with permutations = 4999 was conducted to test for significance. The forward selection of variables was conducted to select the driving variables of assemblage of forest communities as well as to explain the variation in species with highly correlated variables. Monte Carlo test was applied to prove the significant correlation with 4999 permutations. Plot × species matrices and the estimation of IV were conducted in Microsoft Office Excel and all the statistical analyses were processed in RStudio software (version 4.2.0) at a 95% confidence level.

## 5. Conclusions

The present study revealed that there are four major evergreen forest communities, i.e., *Vatica maingayi* forest (VMF), *Quercus glauca* forest (QGF), *Nothaphoebe condensa* forest (NCF), and *Diospyros toposia* forest (DTF), and one minor forest community, i.e., bamboo forest (BF), in Htamanthi Wildlife Sanctuary (HWS). Based on Shannon diversity index estimation, three of the five communities have high to very high species diversity, while the other two communities have low to moderate species diversity. Here, the soil Ca, Mg, clay proportion, soil hardness, and elevation were identified as the major factors in classifying the forest community types. Identification of these community types and understanding of the major factors influencing their structure is an important nature-based solution in biodiversity conservation for a biodiversity hotspot like HWS. The present work will enhance our understanding on how to efficiently implement an ecosystem-based conservation approach through conservation prioritization.

## Figures and Tables

**Table 1:** Species diversity values among forest community types in Htamanthi Wildlife Sanctuary in Myanmar. Values in parenthesis are the importance values of the dominant tree species.

Forest Communities	Richness (S)	Shannon-Wiener Diversity Index (H)	Evenness (J′)	Dominant Species and Importance Values (IV)
**BF**	10	0.086	0.037	*Aglaia perviridis* (17.3%)
**DTF**	51	2.659	0.676	*Diospyros toposia* (25.2%)
**NCF**	95	2.807	0.616	*Nothaphoebe condensa* (26.2%)
**QGF**	65	3.254	0.780	*Quercus glauca* (30.9%)
**VMF**	174	3.965	0.769	*Vatica maingayi* (35.9%)

**Table 2:** Topographic factors in the identified forest community types in Htamanthi Wildlife Sanctuary in Myanmar. Values in parenthesis are the standard deviations from the mean. Different lowercase letters indicate statistical significance between community types at α = 0.05.

FC	n	Elevation (masl)	Slope (°)
DTF	5	274 (57) ^a^	29 (19) ^a^
NCF	10	188 (32) ^ab^	8 (6) ^b^
QGF	13	179 (24) ^b^	16 (12) ^ab^
VMF	36	177 (31) ^b^	16 (14) ^ab^

**Table 3:** Soil physical properties in the *Diospyros toposia* forest (DTF), *Nothaphoebe condensa* forest (NCF), *Quercus glauca* forest (QGF), and *Vatica maingayi* forest (VMF). Values in parenthesis are the standard deviations from the mean. Different lowercase letters indicate statistical significance between forest community types at α = 0.05. Values in parenthesis are the standard deviations from the mean.

Soil Depth (cm)	Forest Community	n	Soil Hardness (kg/cm^2^)	Moisture Content (%)	Bulk Density (%)	Organic Matter (%)	Sand (%)	Silt (%)	Clay (%)
0–15	DTF	5	2.50 (0.55) ^a^	10.30 (3.19) ^a^	1.18 (0.06) ^a^	6.00 (1.87) ^a^	54.2 (4.2) ^c^	29.0 (4.2) ^a^	16.2 (3.5) ^a^
	NCF	10	2.79 (0.88) ^a^	20.10 (4.32) ^b^	1.18 (0.14) ^a^	7.30 (2.11) ^a^	39.4 (13.1) ^a^	30.3 (7.6) ^a^	29.2 (7.3) ^b^
	QGF	13	3.31 (0.72) ^a^	17.89 (8.04) ^ab^	1.10 (0.09) ^a^	6.69 (1.25) ^a^	50.0 (5.3) ^bc^	29.8 (3.7) ^a^	19.1 (3.8) ^a^
	VMF	36	3.00 (1.11) ^a^	20.10 (9.80) ^b^	1.19 (0.14) ^a^	6.00 (1.87) ^a^	46.6 (6.7) ^ab^	27.6 (5.0) ^a^	24.5 (6.1) ^b^
15–30	DTF	5	3.06 (1.18) ^a^	12.00 (2.95) ^a^	1.13 (0.09) ^a^	6.20 (1.48) ^a^	46.8 (4.3) ^a^	27.0 (3.2) ^a^	22.6 (3.0) ^a^
	NCF	10	2.89 (0.87) ^a^	21.80 (3.50) ^b^	1.16 (0.11) ^a^	7.70 (1.34) ^a^	42.1 (2.5) ^a^	28.9 (8.2) ^a^	32.6 (5.9) ^b^
	QGF	13	3.94 (0.48) ^b^	16.30 (2.52) ^a^	1.05 (0.10) ^a^	7.15 (1.14) ^a^	46.2 (2.2) ^a^	28.8 (3.7) ^a^	23.1 (4.3) ^a^
	VMF	36	3.05 (1.02) ^ab^	19.80 (4.63) ^b^	1.13 (0.09) ^a^	6.97 (1.13) ^a^	42.3 (1.3) ^a^	26.6 (4.8) ^a^	27.9 (6.8) ^ab^

**Table 4:** Soil chemical properties in the *Diospyros toposia* forest (DTF), *Nothaphoebe condensa* forest (NCF), *Quercus glauca* forest (QGF), and *Vatica maingayi* forest (VMF). Values in parenthesis are the standard deviations from the mean. Different lowercase letters indicate statistical significance between forest community types at α = 0.05. Values in parenthesis are the standard deviations from the mean. Abbreviations: TN—total nitrogen, AP—available phosphorus, K—extractable potassium, Ca—extractable calcium, Na—extractable sodium, Mg—extractable magnesium.

Soil Depth (cm)	Forest Community	n	pH	TN (g/kg)	AP (mg/kg)	K (mg/100 g)	Ca (mg/100 g)	Na (mg/100 g)	Mg (mg/100 g)
0–15	DTF	5	5.12 (0.19) ^a^	0.590 (0.136) ^a^	80.0 (7.10) ^a^	8.40 (3.78) ^a^	63.80 (6.44) ^a^	0.30 (0.07) ^a^	154.40 (144.0) ^a^
	NCF	10	4.94 (0.09) ^a^	0.657 (0.285) ^a^	47.0 (35.0) ^b^	5.20 (3.52) ^a^	10.20 (6.25) ^b^	0.32 (0.09) ^a^	21.60 (28.7) ^ab^
	QGF	13	4.85 (0.23) ^a^	0.611 (0.189) ^a^	55.4 (13.9) ^b^	4.15 (1.57) ^a^	5.15 (3.63) ^b^	0.22 (0.15) ^a^	18.77 (18.2) ^ab^
	VMF	36	4.89 (0.11) ^a^	0.663 (0.141) ^a^	50.0 (20.6) ^b^	5.14 (3.09) ^a^	7.36 (8.59) ^b^	0.28 (0.14) ^a^	12.19 (18.0) ^b^
15–30	DTF	5	4.98 (0.12) ^a^	0.622 (0.955) ^a^	80.0 (21.5) ^a^	8.80 (4.32) ^a^	13.60 (12.60) ^a^	0.24 (0.11) ^a^	72.40 (49.0) ^a^
	NCF	10	4.97 (0.09) ^a^	0.572 (0.174) ^a^	44.0 (32.4) ^b^	5.00 (3.56) ^a^	7.60 (3.92) ^ab^	0.34 (0.18) ^a^	19.80 (29.8) ^b^
	QGF	13	5.08 (0.84) ^a^	0.600 (0.129) ^a^	45.4 (32.4) ^b^	4.08 (1.93) ^a^	3.15 (2.61) ^b^	0.19 (0.13) ^a^	19.46 (21.6) ^b^
	VMF	36	4.94 (0.13) ^a^	0.618 (0.172) ^a^	42.8 (16.1) ^b^	5.28 (3.09) ^a^	6.39 (7.53) ^ab^	0.26 (0.16) ^a^	19.19 (54.7) ^b^

**Table 5:** Ecological/biotic parameters measured in Htamanthi Wildlife Sanctuary. Abbreviations: **SDen**—species density; **RDen**—relative density; **SFre**—species frequency; **RFre**—relative frequency; **SDom**—species dominance; **RDom**—relative dominance; **IV**—importance values; **BA**—basal area; **AS**—area sampled; DBH—diameter at breast height.

Parameters	Description	Formula
Species density	The actual size or number of individuals of one species per unit area.	Den = No. of individuals of each species/AS
Relative density	The density of one species as a percent of the total density of all species.	RDen = (Den for a species/total density for all identified species) × 100
Species frequency	The number of times a plant species is present in a given number of plots or quadrats.	Fre = No. of plots in which species occur/ total number of plots sampled
Relative frequency	The frequency of one species as a percent of the total frequency of all species.	RFre = (Fre for a species/total frequency for all identified species) × 100
Dominance	A species that is most commonly found or dominant based on basal area or percent coverage.	Dom = BA of one species/AS
Relative dominance	The dominance of one species as a percent of the total dominance of all species.	RDom = (Dom for a species/total dominance for all identified species) × 100
Importance values	The IV is a measure of how dominant a species is in a given forest area.	IV = RDen + RFre + RDom

## Data Availability

The data used are primarily reflected in the article. Other relevant data are available from the authors upon request.

## Supplementary Materials

The following supporting information can be downloaded at: https://www.mdpi.com/article/10.3390/plants11162180/s1, Table S1: List of tree species surveyed in Htamanthi Wildlife Sanctuary; Table S2: *p* values estimated by Kruskal–Wallis test for soil physical properties across soil depths and forest communities; Table S3: *p* values estimated by Kruskal–Wallis test for soil chemical properties across soil depths and forest communities; Table S4: Significance of test of the global model with all explanatory variables of topographic factors and soil properties; Table S5: Selection of important variables of topographic factors and soil properties at 0–15 cm depth of soil; Table S6: Selection of important variables of topographic factors and soil properties at 15–30 cm depth of soil.
