# Supplementary material for: Tree Species Composition and Forest Community Types along Environmental Gradients in Htamanthi Wildlife Sanctuary, Myanmar: Implications for Action Prioritization in Conservation
Source: Plants (Basel). 2022 Aug 22;11(16):2180. doi: 10.3390/plants11162180 (PMC9414654; doi:10.3390/plants11162180)
Supplement: Supplementary file 1 [file plants-11-02180-s001.zip › plants-1759488-supplementary.pdf]

Supplementary Materials

Table S1: List of tree species surveyed in Htamanthi Wildlife Sanctuary.

Community Type: Bamboo Forest (BF)

SCIENTIFIC NAME	RDEN	RDOM	RFREQ	IVI
**ANACARDIACEAE**				
Mangifera indica	0.024	0.000	0.1000	0.124
Spondias pinnata	0.024	0.004	0.1000	0.129
**FABACEAE**				
Albizia chinensis	0.024	0.009	0.1000	0.133
**LYTHRACEAE**				
Duabanga grandiflora	0.049	0.004	0.1000	0.153
**MALVACEAE**				
Stereospermum colais	0.024	0.003	0.1000	0.127
**MELIACEAE**				
Dysoxylum acutangulum	0.024	0.000	0.1000	0.124
Aglaia perviridis	0.073	0.000	0.1000	0.173
**MYRISTICACEAE**				
Knema glauca	0.024	0.000	0.1000	0.124
**POACEAE**				
Dendrocalamus hamiltonii	0.732	0.980	0.2000	1.912
**Total**	**1.00**	**1.00**	**1.00**	**3.00**

Community Type: *Diospyros toposia* forest (DTF)

SCIENTIFIC NAME	RDEN	RDOM	RFREQ	IVI
**ANACARDIACEAE**				
Mangifera indica	0.016	0.023	0.033	0.072
**ANNONACEAE**				
Polyalthia crassa	0.011	0.000	0.011	0.022
**APOCYNACEAE**				
Alstonia rostrata	0.011	0.019	0.022	0.051
**CALOPHYLLACEAE**				
Mesua ferrea	0.095	0.018	0.033	0.146
**COMBRETACEAE**				
Terminalia citrina	0.005	0.001	0.011	0.017
**DILLENIACEAE**				
Tetracera sarmentosa	0.005	0.000	0.011	0.016
**DIPTERCARPACEAE**				
Dipterocarpus baudii	0.011	0.001	0.011	0.022
Dipterocarpus retusus	0.079	0.070	0.044	0.193
Vatica maingayi	0.047	0.017	0.044	0.108
**EBENACEAE**				
Diospyros toposia	0.147	0.049	0.055	0.252
**EUPHOBIACEAE**				
Balakata baccata	0.011	0.004	0.011	0.025

SCIENTIFIC NAME	RDEN	RDOM	RFREQ	IVI
Chrozophora plicata	0.005	0.000	0.011	0.016
Triadica cochinchinensis	0.011	0.009	0.011	0.031
Croton sp. 23	0.005	0.000	0.011	0.016
**FABACEAE**				
Albizia chinensis	0.005	0.007	0.011	0.024
Saraca indica	0.026	0.003	0.044	0.073
Saraca sp.	0.005	0.000	0.011	0.016
**FAGACEAE**				
Castanopsis polystachyus	0.032	0.014	0.022	0.068
Castanopsis sp.2	0.005	0.000	0.011	0.016
Castanopsis tribuloides	0.026	0.027	0.033	0.086
Lithocarpus dealbatus	0.026	0.017	0.022	0.066
Quercus glauca	0.005	0.001	0.011	0.017
Quercus semiserrata	0.016	0.001	0.011	0.028
**IXONANTHACEAE**				
Ixonanthes chinensis	0.032	0.027	0.022	0.080
**LAMIACEAE**				
Tectona grandis	0.047	0.107	0.022	0.177
Vitex sp. 2	0.005	0.000	0.011	0.016
**LAURACEA**				
Nothaphoebe condensa	0.011	0.002	0.022	0.035
Actinodaphne bourdilonii	0.005	0.000	0.011	0.016
Actinodaphne sp.2	0.005	0.001	0.011	0.017
Actinodaphne sp.3	0.005	0.001	0.011	0.017
**LOGANIACEAE**				
Strychnos wallichiana	0.011	0.001	0.011	0.022
**MAGNOLIACEAE**				
Magnolia sp.3	0.011	0.005	0.011	0.027
**MALVACEAE**				
Hibiscus macrophyllus	0.011	0.002	0.022	0.035
Pterospermum javanicum	0.005	0.016	0.011	0.032
**MELIACEAE**				
Cedrela sp.	0.005	0.008	0.011	0.024
Aglaia perviridis	0.011	0.007	0.022	0.039
Chisocheton cumingianus	0.011	0.005	0.022	0.038
**MYRISTICACEAE**				
Horsfieldia glabra	0.011	0.003	0.011	0.025
Knema glauca	0.005	0.000	0.011	0.016
**MYRTACEAE**				
Sysygium sp. 1	0.011	0.008	0.011	0.030
Sysygium sp. 4	0.005	0.002	0.011	0.018
Sysygium sp. 5	0.011	0.004	0.011	0.025
**OLACACEAE**				
Anacolosa sp. 2	0.011	0.003	0.022	0.035
**PHYLLANTHACEAE**				

SCIENTIFIC NAME	RDEN	RDOM	RFREQ	IVI
Baccaurea ramiflora	0.021	0.003	0.022	0.046
Phyllanthus emblica	0.011	0.003	0.022	0.036
Antidesma sp. 31	0.005	0.000	0.011	0.016
Aporosa roxburghii	0.032	0.003	0.033	0.067
Aporosa sp. 63	0.005	0.000	0.011	0.016
**POACEAE**				
Dendrocalamus hamiltonii	0.047	0.498	0.033	0.578
**POLYGALACEAE**				
Xanthophyllum flavescens	0.005	0.002	0.011	0.018
**ROSACEAE**				
Eribotrya sp.	0.011	0.002	0.011	0.023
**RUBIACEAE**				
Wendlandia sp. 1	0.011	0.001	0.022	0.034
**RUTACEAE**				
Aegle marmelos	0.005	0.000	0.011	0.017
**SALICACEAE**				
Flacourtia inermis	0.011	0.004	0.011	0.025
**SYMPLOCACEAE**				
Symplocos sp.1	0.005	0.001	0.011	0.017
**Total**	**1.00**	**1.00**	**1.00**	**3.00**

Community Type: ***Nothaphoebe condensa*** **forest (NCF)**

SCIENTIFIC NAME	RDEN	RDOM	RFREQ	IVI
**ACHARIACEAE**				
Hydnocarpus kurzii	0.046	0.009	0.025	0.080
**ANACARDIACEAE**				
Buchanania arborescens	0.005	0.000	0.004	0.009
Mangifera indica	0.017	0.052	0.021	0.090
Holigarna helferi	0.009	0.004	0.017	0.030
**ANNONACEAE**				
Polyalthia simiarum	0.005	0.000	0.008	0.013
Polyalthia sp.1	0.002	0.000	0.004	0.006
Polyalthia sp.2	0.003	0.000	0.004	0.008
**APOCYNACEAE**				
Alstonia rostrata	0.003	0.000	0.008	0.012
Alstonia scholaris	0.002	0.000	0.004	0.006
**ARALIACEAE**				
Heteropanax fragrans	0.002	0.000	0.004	0.006
**ARECACEAE**				
Caryota sp.	0.003	0.001	0.004	0.008
Livistona jenkinsiana	0.029	0.021	0.030	0.080
**BURSERACEAE**				
Canarium sp.1	0.028	0.010	0.025	0.063
Canarium denticulatum	0.003	0.005	0.004	0.012

SCIENTIFIC NAME	RDEN	RDOM	RFREQ	IVI
**CALOPHYLLACEAE**				
Mesua ferrea	0.055	0.014	0.038	0.107
**CLUSIACEAE**				
Garcinia cowa	0.018	0.003	0.008	0.030
Garcinia elliptica	0.011	0.002	0.021	0.034
Garcinia xanthochymus	0.018	0.002	0.017	0.037
**COMBRETACEAE**				
Terminalia bellerica	0.002	0.001	0.004	0.006
Terminalia chebula	0.005	0.005	0.008	0.018
Terminalia sp.2	0.003	0.001	0.004	0.009
**DILLENIACEAE**				
Dillenia pentagyna	0.005	0.005	0.013	0.022
**DIPTERCARPACEAE**				
Dipterocarpus retusus	0.005	0.002	0.013	0.019
Vatica maingayi	0.054	0.047	0.008	0.109
**EBENACEAE**				
Diospyros toposia	0.046	0.013	0.030	0.089
**EBENACEAE**				
Mallotus paniculatus	0.005	0.000	0.008	0.013
Mallotus philippinensis	0.002	0.000	0.004	0.006
**FABACEAE**				
Saraca indica	0.003	0.000	0.008	0.012
Castanopsis indica	0.006	0.002	0.008	0.017
Castanopsis polystachyus	0.003	0.000	0.008	0.012
Castanopsis tribuloides	0.006	0.002	0.013	0.021
Lithocarpus dealbatus	0.011	0.017	0.013	0.040
Lithocarpus fenestrata	0.005	0.002	0.008	0.015
Lithocarpus sp.1	0.002	0.001	0.004	0.007
Quercus glauca	0.006	0.003	0.004	0.013
Quercus semiserrata	0.009	0.004	0.008	0.022
**LAMIACEAE**				
Vitex sp. 1	0.002	0.000	0.030	0.031
**LAURACEA**				
Nothaphoebe condensa	0.206	0.039	0.038	0.282
Phoebe sp.1	0.002	0.000	0.004	0.006
Actinodaphne bourdilonii	0.005	0.000	0.013	0.017
Actinodaphne sp.3	0.002	0.000	0.004	0.006
Cinnamomum nitidum	0.002	0.000	0.004	0.006
Litsea sp.	0.002	0.002	0.004	0.008
**LYTHRACEAE**				
Duabanga grandiflora	0.005	0.001	0.008	0.014
**MAGNOLIACEAE**				
Magnolia macrophylla	0.002	0.000	0.004	0.006
Magnolia praecalva	0.011	0.001	0.013	0.024
Magnolia sp.1	0.003	0.001	0.004	0.008

SCIENTIFIC NAME	RDEN	RDOM	RFREQ	IVI
Magnolia sp.2	0.005	0.005	0.008	0.018
Magnolia sp.3	0.002	0.010	0.004	0.015
**MALVACEAE**				
Bombax insigne	0.002	0.013	0.004	0.019
Pterospermum aceroides	0.002	0.000	0.004	0.006
Pterospermum javanicum	0.002	0.002	0.004	0.008
Pterospermum semisagittatum	0.002	0.001	0.004	0.007
Stereospermum colais	0.002	0.001	0.004	0.007
**MELIACEAE**				
Dysoxylum acutangulum	0.006	0.004	0.013	0.023
Dysoxylum excelsum	0.002	0.000	0.004	0.006
Aglaia perviridis	0.008	0.001	0.017	0.026
Chisocheton cumingianus	0.014	0.002	0.021	0.037
Chisocheton cumingianus subsp. balansae	0.003	0.001	0.004	0.009
**MORACEAE**				
Antiaris toxicaria	0.002	0.000	0.004	0.006
Artocarpus lacucha	0.003	0.005	0.008	0.016
Ficus sp. 1	0.002	0.001	0.004	0.006
Ficus sp. 6	0.002	0.001	0.004	0.006
**MYRISTICACEAE**				
Knema furfuraceae	0.002	0.000	0.004	0.006
Knema glauca	0.029	0.003	0.034	0.066
Knema globularia	0.011	0.003	0.013	0.026
Knema linifolia	0.003	0.001	0.008	0.012
**MYRTACEAE**				
Sysygium diospyrifolium	0.003	0.000	0.004	0.008
Sysygium megacarpum	0.002	0.001	0.004	0.007
Sysygium sp. 1	0.011	0.006	0.013	0.029
Sysygium sp. 10	0.002	0.000	0.004	0.006
Sysygium sp. 4	0.006	0.001	0.008	0.016
Sysygium sp. 8	0.002	0.003	0.004	0.008
**OLACACEAE**				
Anacolosa sp. 1	0.006	0.001	0.008	0.016
Chionanthus ramiflorus	0.002	0.000	0.004	0.006
**PANDACEAE**				
Galearia filiformis	0.006	0.000	0.013	0.019
**PHYLLANTHACEAE**				
Baccaurea ramiflora	0.008	0.001	0.017	0.025
Aporosa roxburghii	0.023	0.002	0.021	0.046
**POACEAE**				
Dendrocalamus longispathus	0.040	0.527	0.021	0.588
Pseudostachyum polymorphum	0.020	0.093	0.004	0.117
**POLYGALACEAE**				
Xanthophyllum flavescens	0.025	0.005	0.013	0.043

SCIENTIFIC NAME	RDEN	RDOM	RFREQ	IVI
**RHIZOPHORACEAE**				
Carallia brachiata	0.005	0.000	0.008	0.013
**ROSACEAE**				
Eribotrya bengalensis	0.002	0.001	0.004	0.007
Eribotrya sp.	0.002	0.001	0.004	0.006
**RUBIACEAE**				
Psydrax sp.	0.003	0.001	0.008	0.012
Wendlandia sp. 1	0.009	0.002	0.004	0.015
**RUTACEAE**				
Acronychia sp.	0.003	0.001	0.004	0.008
Glycosmis sp. 1	0.002	0.000	0.004	0.006
**SALICACEAE**				
Casearia sp.	0.003	0.000	0.004	0.008
Flacourtia inermis	0.003	0.000	0.008	0.012
**SAPINDACEAE**				
Xerospermum noronhianum	0.028	0.021	0.021	0.069
**SYMPLOCACEAE**				
Symplocos recemosa	0.008	0.000	0.013	0.021
Symplocos sp.1	0.006	0.003	0.013	0.021
Symplocos sp.2	0.002	0.000	0.004	0.006
**VITACEAE**				
Leea macrophylla	0.002	0.000	0.004	0.006
**UNIDENTIFIED**	0.003	0.000	0.038	0.042
**Total**	**1.00**	**1.00**	**1.00**	**3.00**

Community Type: *Quercus glauca* forest (QGF)

SCIENTIFIC NAME	RDEN	RDOM	RFREQ	IVI
**ACHARIACEAE**				
Hydnocarpus kurzii	0.024	0.012	0.034	0.070
**ANACARDIACEAE**				
Gluta sp.	0.006	0.009	0.015	0.030
Mangifera indica	0.001	0.001	0.005	0.007
Holigarna helferi	0.007	0.028	0.020	0.055
**ANNONACEAE**				
Goniothalamus sp. 1	0.001	0.000	0.005	0.006
Polyalthia crassa	0.009	0.005	0.020	0.034
Polyalthia hookeriana	0.001	0.001	0.005	0.007
Polyalthia sp.1	0.039	0.038	0.015	0.091
**APOCYNACEAE**				
Alstonia rostrata	0.012	0.022	0.015	0.049
Holarrhena pubescens	0.003	0.002	0.010	0.015
**BURSERACEAE**				

SCIENTIFIC NAME	RDEN	RDOM	RFREQ	IVI
Canarium sp.1	0.001	0.005	0.005	0.011
**CALOPHYLLACEAE**				
Mesua ferrea	0.121	0.064	0.054	0.239
**CLUSIACEAE**				
Garcinia pedunculata	0.024	0.009	0.039	0.072
Garcinia xanthochymus	0.006	0.002	0.010	0.017
**DILLENIACEAE**				
Dillenia pentagyna	0.006	0.009	0.010	0.025
**DIPTERCARPACEAE**				
Dipterocarpus kerrii	0.004	0.041	0.005	0.050
Dipterocarpus retusus	0.051	0.171	0.029	0.252
Vatica maingayi	0.060	0.093	0.039	0.192
**EBENACEAE**				
Diospyros toposia	0.085	0.044	0.054	0.183
**EUPHORBIACEAE**				
Macaranga denticulata	0.004	0.000	0.010	0.015
**FABACEAE**				
Cynometra sp.	0.003	0.004	0.010	0.016
Saraca indica	0.007	0.001	0.010	0.019
Saraca sp.	0.001	0.001	0.005	0.007
Xylia xylocarpa	0.001	0.001	0.005	0.007
Castanopsis tribuloides	0.004	0.004	0.010	0.018
Lithocarpus elegans	0.003	0.001	0.005	0.008
Lithocarpus fenestrata	0.004	0.003	0.010	0.017
Quercus glauca	0.174	0.086	0.049	0.309
**IXONANTHACEAE**				
Ixonanthes chinensis	0.004	0.013	0.015	0.032
**LAURACEAE**				
Alseodaphne nigrescens	0.003	0.005	0.010	0.017
Endiandra sp.	0.001	0.005	0.005	0.011
Neolitsea zeylanica	0.003	0.003	0.005	0.011
**LOGANIACEAE**				
Strychnos nux-blanda	0.001	0.001	0.005	0.007
**LYTHRACEAE**				
Duabanga grandiflora	0.045	0.010	0.039	0.094
Lagerstroemia sp.	0.001	0.000	0.005	0.007
**MAGNOLIACEAE**				
Magnolia macrophylla	0.001	0.000	0.005	0.007
Magnolia sp.1	0.013	0.024	0.010	0.047
Magnolia sp.3	0.003	0.003	0.005	0.011
**MALVACEAE**				
Pterospermum javanicum	0.019	0.031	0.020	0.070

SCIENTIFIC NAME	RDEN	RDOM	RFREQ	IVI
Sterculia macrophylla	0.004	0.002	0.010	0.016
Stereospermum colais	0.001	0.004	0.005	0.010
**MELIACEAE**				
Dysoxylum acutangulum	0.004	0.002	0.010	0.017
Chisocheton cumingianus	0.003	0.018	0.005	0.026
Chisocheton cumingianus subsp. balansae	0.001	0.004	0.005	0.010
**MORACEAE**				
Ficus sp.2	0.003	0.001	0.005	0.009
**MYRISTICACEAE**				
Horsfieldia glabra	0.001	0.001	0.005	0.007
Knema glauca	0.052	0.010	0.059	0.121
**MYRTACEAE**				
Sysygium sp. 1	0.022	0.018	0.044	0.085
Sysygium sp. 2	0.004	0.001	0.010	0.016
Sysygium sp. 4	0.003	0.001	0.005	0.009
**OLACACEAE**				
Anacolosa sp. 2	0.006	0.003	0.010	0.018
**PANDACEAE**				
Galearia filiformis	0.003	0.000	0.005	0.008
**PHYLLANTHACEAE**				
Baccaurea ramiflora	0.015	0.004	0.034	0.054
Aporosa roxburghii	0.013	0.004	0.025	0.042
**PITTOSPORANCEAE**				
Pittosporum sp.	0.004	0.003	0.015	0.022
**POACEAE**				
Dendrocalamus hamiltonii	0.001	0.085	0.005	0.092
**POLYGALACEAE**				
Xanthophyllum flavescens	0.016	0.011	0.025	0.052
**ROSACEAE**				
Eribotrya bengalensis	0.003	0.001	0.005	0.009
**RUTACEAE**				
Aegle marmelos	0.004	0.003	0.010	0.018
Atalantia sp.	0.010	0.007	0.010	0.027
**SAPINDACEAE**				
Xerospermum noronhianum	0.040	0.058	0.049	0.148
**ULMACEAE**				
Gironniera subaequalis	0.001	0.000	0.005	0.007
Unidentified	0.013	0.008	0.020	0.042
**Total**	**1.00**	**1.00**	**1.00**	**3.00**

Community Type: ***Vatica maingayi*** **forest (VMF)**

SCIENTIFIC NAME	RDEN	RDOM	RFREQ	IVI
**ACHARIACEAE**				
Hydnocarpus kurzii	0.043	0.022	0.028	0.093

SCIENTIFIC NAME	RDEN	RDOM	RFREQ	IVI
Hydnocarpus macrocarpa	0.010	0.005	0.010	0.025
Hydnocarpus sp.	0.000	0.001	0.001	0.003
**ANACARDIACEAE**				
Buchanania arborescens	0.003	0.001	0.005	0.008
Gluta sp.	0.001	0.000	0.002	0.004
Mangifera indica	0.005	0.007	0.005	0.017
Mangifera sp.	0.001	0.000	0.002	0.003
Spondias pinnata	0.000	0.001	0.001	0.003
Holigarna helferi	0.016	0.022	0.019	0.058
**ANNONACEAE**				
Goniothalamus sp. 2	0.000	0.001	0.001	0.002
Polyalthia crassa	0.004	0.001	0.010	0.015
Polyalthia hookeriana	0.004	0.009	0.007	0.020
Polyalthia simiarum	0.009	0.002	0.010	0.021
Polyalthia sp.1	0.042	0.064	0.014	0.120
Polyalthia sp.2	0.003	0.000	0.001	0.004
**APOCYNACEAE**				
Alstonia rostrata	0.004	0.003	0.010	0.017
Alstonia scholaris	0.001	0.000	0.002	0.004
**ARALIACEAE**				
Heteropanax fragrans	0.000	0.000	0.001	0.002
Macropanax dispermus	0.001	0.000	0.001	0.003
Trevesia palmata	0.001	0.000	0.001	0.002
**ARECACEAE**				
Areca triandra	0.002	0.000	0.002	0.005
Livistona jenkinsiana	0.019	0.038	0.018	0.075
**BURSERACEAE**				
Canarium sp.1	0.007	0.004	0.011	0.022
Canarium denticulatum	0.000	0.000	0.001	0.002
**CALOPHYLLACEAE**				
Mesua ferrea	0.102	0.065	0.041	0.208
**CLUSIACEAE**				
Garcinia cowa	0.032	0.008	0.028	0.067
Garcinia elliptica	0.005	0.001	0.007	0.013
Garcinia pedunculata	0.013	0.006	0.013	0.031
Garcinia xanthochymus	0.014	0.004	0.017	0.035
**COMBRETACEAE**				
Terminalia chebula	0.002	0.001	0.006	0.009
Terminalia citrina	0.001	0.008	0.001	0.010
Terminalia sp.2	0.000	0.001	0.001	0.003
**CRYPTERONIACEAE**				
Crypteronia paniculata	0.006	0.024	0.008	0.039
Cyatheaceae				
Cyathea sp.	0.001	0.000	0.001	0.003
**DILLENIACEAE**				

SCIENTIFIC NAME	RDEN	RDOM	RFREQ	IVI
Dillenia pentagyna	0.005	0.025	0.007	0.037
**DIPTERCARPACEAE**				
Dipterocarpus baudii	0.002	0.001	0.002	0.005
Dipterocarpus retusus	0.014	0.015	0.013	0.042
Hopea oblongifolia	0.000	0.000	0.001	0.002
Vatica maingayi	0.115	0.202	0.042	0.359
**EBENACEAE**				
Diospyros apliculata	0.000	0.000	0.001	0.002
Diospyros sp. 2	0.008	0.005	0.012	0.025
Diospyros sp.1	0.001	0.001	0.004	0.006
Diospyros toposia	0.029	0.017	0.028	0.073
**ELAEOCARPACEAE**				
Elaeocarpus sp.	0.000	0.000	0.001	0.002
Sloanea sigun	0.000	0.000	0.001	0.002
**ERICACEAE**				
Lyonia sp.	0.000	0.000	0.001	0.002
**EUPHOBIACEAE**				
Balakata baccata	0.001	0.003	0.002	0.006
Chrozophora plicata	0.002	0.000	0.005	0.008
Croton sp. 23	0.000	0.000	0.001	0.002
Croton sp.1	0.000	0.000	0.001	0.002
**FABACEAE**				
Albizia odoratissima	0.000	0.000	0.001	0.002
Castanospermum australe	0.000	0.000	0.001	0.002
Cynometra sp.	0.015	0.028	0.017	0.060
Saraca indica	0.007	0.001	0.007	0.015
**FAGACEAE**				
Castanopsis indica	0.004	0.001	0.006	0.010
Castanopsis polystachyus	0.009	0.011	0.012	0.032
Castanopsis sp.1	0.001	0.001	0.002	0.004
Castanopsis tribuloides	0.002	0.003	0.004	0.009
Lithocarpus dealbatus	0.000	0.000	0.001	0.002
Lithocarpus elegans	0.000	0.000	0.001	0.002
Lithocarpus fenestrata	0.003	0.002	0.007	0.012
Lithocarpus sp.1	0.002	0.000	0.001	0.004
Quercus helferiana	0.001	0.000	0.001	0.003
Quercus semiserrata	0.004	0.001	0.005	0.009
**GIRONNIERA**				
Gironniera subaequalis	0.004	0.011	0.007	0.022
**IXONANTHACEAE**				
Ixonanthes chinensis	0.009	0.033	0.011	0.053
**LAMIACEAE**				
Callicarpa tomentosa	0.000	0.001	0.001	0.003
**LAURACEA**				
Beilschmiedia sp. 1	0.000	0.001	0.001	0.003

SCIENTIFIC NAME	RDEN	RDOM	RFREQ	IVI
Beilschmiedia sp. 2	0.000	0.000	0.001	0.002
Nothaphoebe condensa	0.086	0.040	0.030	0.156
Phoebe lanceolata	0.003	0.003	0.006	0.012
Phoebe sp. 4	0.000	0.000	0.001	0.002
Phoebe sp.1	0.002	0.002	0.005	0.008
Phoebe sp. 33	0.000	0.000	0.001	0.002
Actinodaphne bourdilonii	0.008	0.002	0.011	0.021
Actinodaphne sp.1	0.001	0.000	0.004	0.006
Actinodaphne sp.3	0.008	0.003	0.012	0.023
Actinodaphne sp.4	0.001	0.000	0.001	0.002
Actinodaphne sp.5	0.001	0.000	0.002	0.004
Alseodaphne nigrescens	0.002	0.000	0.004	0.006
Cinnamomum nitidum	0.002	0.000	0.005	0.007
Cinnamomum sp.	0.001	0.002	0.002	0.006
Endiandra sp.	0.008	0.004	0.005	0.016
Litsea sp.	0.001	0.000	0.002	0.003
**LECYTHIDACEAE**				
Barringtonia macrocarpa	0.000	0.000	0.001	0.002
Barringtonia macrostachya	0.001	0.000	0.002	0.004
**LOGANIACEAE**				
Strychnos potatorum	0.000	0.001	0.001	0.002
**MAGNOLIACEAE**				
Magnolia praecalva	0.005	0.001	0.006	0.012
Magnolia sp.1	0.005	0.004	0.007	0.016
Magnolia sp.2	0.001	0.012	0.002	0.016
Magnolia sp.3	0.000	0.000	0.001	0.002
**MALVACEAE**				
Pterospermum aceroides	0.003	0.006	0.004	0.013
Pterospermum diversifolium	0.007	0.012	0.010	0.029
Pterospermum javanicum	0.005	0.008	0.008	0.021
Pterygota alata	0.001	0.017	0.004	0.022
Sterculia macrophylla	0.004	0.005	0.010	0.019
Stereospermum colais	0.000	0.000	0.001	0.002
Cedrela sp.	0.000	0.000	0.001	0.002
Dysoxylum acutangulum	0.001	0.001	0.002	0.004
Dysoxylum sp.	0.000	0.000	0.001	0.002
Aglaia perviridis	0.012	0.010	0.018	0.040
Aglaia tomentosa	0.001	0.000	0.002	0.004
Chisocheton cumingianus	0.033	0.025	0.034	0.092
Chisocheton cumingianus subsp. balansae	0.002	0.000	0.006	0.009
**MORACEAE**				
Antiaris toxicaria	0.002	0.002	0.002	0.006
Ficus sp. 1	0.000	0.000	0.001	0.002
Ficus sp.2	0.001	0.016	0.001	0.018
Ficus sp.3	0.000	0.000	0.001	0.002

SCIENTIFIC NAME	RDEN	RDOM	RFREQ	IVI
Ficus sp.9	0.000	0.000	0.001	0.002
**MYRISTICACEAE**				
Horsfieldia glabra	0.003	0.001	0.006	0.010
Knema erratica	0.000	0.000	0.001	0.002
Knema furfuraceae	0.006	0.001	0.010	0.016
Knema glauca	0.022	0.005	0.024	0.050
Knema globularia	0.019	0.004	0.022	0.045
Knema sp. 1	0.000	0.000	0.001	0.002
**MYRTACEAE**				
Sysygium diospyrifolium	0.000	0.000	0.001	0.002
Sysygium megacarpum	0.003	0.003	0.004	0.010
Sysygium sp. 1	0.010	0.005	0.017	0.032
Sysygium sp. 10	0.003	0.003	0.007	0.013
Sysygium sp. 2	0.003	0.001	0.002	0.007
Sysygium sp. 3	0.001	0.002	0.004	0.007
Sysygium sp. 4	0.005	0.002	0.006	0.013
Sysygium sp. 5	0.004	0.001	0.006	0.011
Sysygium sp. 9	0.000	0.001	0.001	0.003
**OLACACEAE**				
Anacolosa sp. 1	0.000	0.000	0.001	0.002
Chionanthus decipiens	0.000	0.000	0.001	0.002
Chionanthus ramiflorus	0.000	0.000	0.001	0.002
**PANDACEAE**				
Galearia filiformis	0.011	0.001	0.019	0.032
**PENTAPHYLACEAE**				
Eurya acuminata	0.000	0.000	0.001	0.002
**PHYLLANTHACEAE**				
Baccaurea ramiflora	0.016	0.003	0.025	0.044
Glochidion sp.	0.001	0.000	0.001	0.002
Glochidion superbum	0.000	0.000	0.001	0.002
Antidesma sp. 31	0.000	0.000	0.001	0.002
Aporosa roxburghii	0.006	0.001	0.008	0.015
Aporosa sp. 63	0.000	0.000	0.001	0.002
**PITTOSPORANCEAE**				
Pittosporum sp.	0.001	0.000	0.004	0.005
**POACEAE**				
Dendrocalamus longispathus	0.009	0.015	0.005	0.029
Pseudostachyum polymorphum	0.002	0.076	0.002	0.080
**PODOCARPACEAE**				
Podocarpus neriifolius	0.001	0.000	0.002	0.003
Podocarpus wallichianus	0.000	0.000	0.001	0.002
**POLYGALACEAE**				
Xanthophyllum flavescens	0.017	0.007	0.016	0.039
**PRIMULACEAE**				
Ardisia sp.3	0.000	0.000	0.001	0.002

SCIENTIFIC NAME	RDEN	RDOM	RFREQ	IVI
**RHIZOPHORACEAE**				
Carallia brachiata	0.002	0.001	0.005	0.008
Carallia sp. 22	0.000	0.001	0.001	0.002
**ROSACEAE**				
Eribotrya bengalensis	0.003	0.001	0.005	0.009
**RUBIACEAE**				
Brachytome sp.	0.001	0.000	0.001	0.002
Gardenia sp.	0.000	0.000	0.001	0.002
Ixora sp.2	0.002	0.001	0.005	0.009
Nauclea orientalis	0.000	0.000	0.001	0.002
Pavetta indica	0.000	0.000	0.001	0.002
Psydrax sp.	0.001	0.001	0.001	0.004
Rennellia sp.2	0.000	0.000	0.001	0.002
Tarennoidea wallichii	0.000	0.000	0.001	0.002
Wendlandia paniculata	0.000	0.000	0.001	0.002
Wendlandia puberula	0.000	0.000	0.001	0.002
**RUTACEAE**				
Atalantia sp.	0.005	0.001	0.007	0.013
Clausena heptaphylla	0.001	0.000	0.001	0.002
Glycosmis sp. 1	0.001	0.000	0.001	0.003
**SALICACEAE**				
Flacourtia inermis	0.000	0.000	0.001	0.002
**SAPINDACEAE**				
Xerospermum noronhianum	0.010	0.012	0.019	0.041
**SAPOTACEAE**				
Palaquium sp.	0.000	0.000	0.001	0.002
**SYMPLOCACEAE**				
Symplocos recemosa	0.002	0.001	0.005	0.008
**TERNSTROEMIACEAE**				
Ternstroemia sp.	0.001	0.002	0.001	0.004
**TETRAMELACEAE**				
Tetrameles nudiflora	0.000	0.001	0.001	0.003
**UNIDENTIFIED**	0.003	0.002	0.005	0.009
**Total**	**1.00**	**1.00**	**1.00**	**3.00**

Table S2: P values estimated by Kruskal-Wallis test for soil physical properties across soil depths and forest communities. Hd – mean hardness of soil, MC – moisture content, BD – bulk density, OM – organic matter, Sand – content of sand, Silt – content of silt, Clay – content of clay. df – degree of freedom and H – Kruskal-Wallis chi-squared, N = 64, df = 3, p-values at α = 0.05.

Physical properties of soil	0 -15 cm depth of soil		15 - 30 cm depth of soil		0 - 30 cm depth of soil (Mean)	
	H	p-value	H	p-value	H	p-value
Hd	3.34	0.342	8.29	**<0.05**	9.87	**<0.05**
MC	15.70	**<0.05**	21.01	**<0.001**	36.13	**<0.001**
BD	7.26	0.064	5.57	0.135	10.21	**<0.05**
OM	2.20	0.532	4.91	0.178	6.27	0.099
Sand	12.04	**<0.05**	7.64	0.054	17.76	**<0.001**
Silt	3.82	0.281	3.26	0.354	6.58	0.087
Clay	19.73	**<0.001**	15.16	**<0.05**	32.18	**<0.001**

Table S3: P values estimated by Kruskal-Wallis test for soil chemical properties across soil depths and forest communities. pH – soil pH, TN – Total nitrogen, Ava. P – Available phosphorus, K – Extractable potassium, Ca – Extractable calcium, Na – Extractable sodium, and Mg – Extractable magnesium. df – degree of freedom and H – Kruskal-Wallis chi-squared, N = 64, df = 3, p-values at α = 0.05.

Chemical properties of soil	0 -15 cm depth of soil		15 - 30 cm depth of soil		0 - 30 cm depth of soil (Mean)	
	H	p-value	H	p-value	H	p-value
pH	7.25	0.064	2.22	0.527	8.21	0.042
TN	2.12	0.548	0.42	0.936	1.79	0.618
Ava. P	13.43	**<0.05**	13.48	**<0.05**	26.52	**<0.001**
K	6.49	0.090	6.38	0.095	13.04	**<0.05**
Ca	12.46	**<0.05**	17.45	**<0.001**	26.52	**<0.001**
Na	4.25	0.235	5.43	0.143	9.63	**<0.05**
Mg	16.71	**<0.001**	11.29	**<0.05**	27.37	**<0.001**

Table S4: Significance of test of the global model with all explanatory variables of topographic factors and soil properties at two layers of soil (0-15 cm depth and 15-30 cm depth) and Monte Carlo permutation test (permutations=4999).

Source of variables	Df	Variance	R^2 Adjust	F	Pr > F
Model (0-15 cm)	17	0.224	0.099	1.40	**<0.001**
Residual (0-15 cm)	48	0.453			
Model (15-30 cm)	17	0.218	0.083	1.35	**<0.001**
Residual (15-30 cm)	48	0.458			

Table S5: Selection of important variables of topographic factors and soil properties at 0-15 cm depth of soil affecting the composition of tree species and variation of forest communities with forward selection at permutation = 4999. Ca – Extractable calcium, Clay – content of clay, Hd – mean hardness of soil, ELV – Elevation, Sand – content of sand, and Mg – Extractable magnesium.

Selected Variables	Order	R^2	R^2 Cumulation	R^2_{Adjust} Cumulation	F	Pr	Pr_{adj}
Ca	15	0.045	0.045	0.030	3.01	0.0002	0.0034**
Clay	10	0.034	0.079	0.049	2.31	0.0002	0.0034**
Hd	4	0.025	0.104	0.060	1.73	0.0030	0.0450*
ELV	1	0.023	0.126	0.069	1.59	0.0068	0.0952
Sand	8	0.021	0.148	0.077	1.54	0.0126	0.1368
Mg	17	0.019	0.169	**0.083**	1.38	0.0494	0.5928

***p < 0.001, **p < 0.01, *p < 0.05

Table S6: Selection of important variables of topographic factors and soil properties at 15-30 cm depth of soil affecting the composition of tree species and variation of forest communities with forward selection at permutation = 4999. Hd – mean hardness of soil, Ca – Extractable calcium, ELV – elevation and BD – bulk density.

Selected Variables	Order	R^2	R^2 Cumulation	R^2_{Adjust} Cumulation	F	Pr	Pr_{adj}
Hd	4	0.035	0.035	0.020	2.31	0.0002	0.0034**
Ca	15	0.033	0.068	0.038	2.20	0.0002	0.0034**
ELV	1	0.034	0.101	0.058	2.32	0.0002	0.0034**
BD	6	0.024	0.125	**0.067**	1.65	0.0058	0.0812

***p < 0.001, **p < 0.01, *p < 0.05
